# Risk factors for COVID-19 mortality: The effect of convalescent plasma administration

**DOI:** 10.1371/journal.pone.0250386

**Published:** 2021-04-29

**Authors:** Martín R. Salazar, Soledad E. González, Lorena Regairaz, Noelia S. Ferrando, Verónica V. González Martínez, Patricia M. Carrera Ramos, Laura Muñoz, Santiago A. Pesci, Juan M. Vidal, Nicolás Kreplak, Elisa Estenssoro

**Affiliations:** 1 Teaching and Research Service, San Martín Hospital, La Plata, Buenos Aires, Argentina; 2 Faculty of Medicine, National University of La Plata, Buenos Aires, Argentina; 3 Ministry of Health of the Province of Buenos Aires, La Plata, Buenos Aires, Argentina; 4 Immunology Unit, Children´s Hospital Sor Maria Ludovica, La Plata, Buenos Aires, Argentina; 5 Hemotherapy Institute of Buenos Aires Province "Dra Nora Etchenique", La Plata, Buenos Aires, Argentina; 6 Pediatric Research Institute "Prof. Fernando E. Vitieri”, Children´s Hospital Sor Maria Ludovica, La Plata, Buenos Aires, Argentina; 7 Intensive Care Unit, San Martín Hospital, La Plata, Buenos Aires, Argentina; University of Florida, UNITED STATES

## Abstract

**Background:**

Convalescent plasma, widely utilized in viral infections that induce neutralizing antibodies, has been proposed for COVID-19, and preliminary evidence shows that it might have beneficial effect. Our objective was to determine the risk factors for 28-days mortality in patients who received convalescent plasma for COVID-19 compared to those who did not, who were admitted to hospitals in Buenos Aires Province, Argentina, throughout the pandemic.

**Methods:**

This is a multicenter, retrospective cohort study of 2-month duration beginning on June 1, 2020, including unselected, consecutive adult patients with diagnosed COVID-19, admitted to 215 hospitals with pneumonia. Epidemiological and clinical variables were registered in the Provincial Hospital Bed Management System. Convalescent plasma was supplied as part of a centralized, expanded access program.

**Results:**

We analyzed 3,529 patients with pneumonia, predominantly male, aged 62±17, with arterial hypertension and diabetes as main comorbidities; 51.4% were admitted to the ward, 27.1% to the Intensive Care Unit (ICU), and 21.7% to the ICU with mechanical ventilation requirement (ICU-MV). 28-day mortality was 34.9%; and was 26.3%, 30.1% and 61.4% for ward, ICU and ICU-MV patients. Convalescent plasma was administered to 868 patients (24.6%); their 28-day mortality was significantly lower (25.5% vs. 38.0%, p<0.001). No major adverse effects occurred. Logistic regression analysis identified age, ICU admission with and without MV requirement, diabetes, and preexistent cardiovascular disease as independent predictors of 28-day mortality, whereas convalescent plasma administration acted as a protective factor.

**Conclusions:**

Our study suggests that the administration of convalescent plasma in COVID-19 pneumonia admitted to the hospital might be associated with improved outcomes.

## Introduction

In December 2019 in Wuhan, China, the first cases of pneumonia caused by SARS-CoV-2, a novel coronavirus, were reported; the disease was subsequently named COVID-19. The new virus spread across the world relentlessly, and on March 11 the World Health Organization declared COVID-19 a pandemic. Up to now, COVID-19 cases are approaching 30,000,000 with 935,000 dead [1, 2].

Few treatments have proven effective for COVID-19 [3]. The administration of convalescent plasma, widely utilized in viral infections that induce neutralizing antibodies, has also been proposed [4–6]. It was used during outbreaks of severe acute respiratory disease caused by other coronaviruses, SARS-CoV-1 and MERS-CoV, with varying results and when administered early, it decreased length of hospital stay [7–9]. Convalescent plasma utilization has an acceptable safety profile and its administration constitutes a feasible approach to implement during a pandemic, even in low-resource settings. In COVID-19, it might reduce viral burden, improve clinical status, and decrease mortality [10–12]. On March 24, 2020, the Food and Drug Administration of the United States launched an Expanded Access Program to collect convalescent plasma donated by individuals who had recovered from COVID-19, and on August 23 approved emergency use [13]. A study conducted in 20,000 patients confirmed the safety of convalescent plasma and, thereafter, in a study of 30,000 patients, the same group of researchers demonstrated a decrease in mortality when convalescent plasma was administered early in the course of COVID-19 [11, 14]. Convalescent plasma is currently being evaluated in 126 clinical trials [15].

Early in the emergency caused by the COVID-19 pandemic, the Ministry of Health of the Province of Buenos Aires, Argentina, created the Centralized Registry of Convalescent Plasma Donors (CROCPD-BA), with the aim of collecting, processing and distributing convalescent plasma, and issuing recommendations for its use in patients with COVID-19 [16]. Accordingly, the objective of the present study was to determine the risk factors for 28-days mortality in patients who received convalescent plasma for COVID-19 and those who did not, who were admitted to hospitals in Buenos Aires Province for COVID-19 throughout the pandemic.

## Materials and methods

This was a multicenter cohort study conducted over 2 months, beginning on June 1, 2020, which included consecutive patients ≥18 years diagnosed with SARS CoV-2 with RT-PCR, admitted to hospitals with pneumonia. Data were obtained from the National Vigilance System (SNVS 2.0), the Provincial Hospital Bed Management System, and the CROCPD-BA.

Collected variables were age, gender, comorbidities [17, 18] (arterial hypertension, diabetes, preexistent cardiovascular disease, chronic obstructive pulmonary disease, immunodeficiency), requirement of mechanical ventilation, treatments, death or discharge, and convalescent plasma administration. Severe adverse events related to plasma infusion, as transfusion-related acute lung injury (TRALI) and transfusion-associated circulatory overload (TACO) were also recorded [19].

Information about plasma collection and characteristics is available in the S1 File.

The requirement of convalescent plasma was initiated by assistant physicians as part of a Program of Expanded Access [16]. The indications issued by the CROCPD-BA were presence of pneumonia, defined as of lung infiltrates, plus one of the following:

- Dyspnea with respiratory rate ≥ 30 breaths/minute
- Oxygen saturation ≤93%
- Oxygen requirement
- PaO_2_FIO_2_<300 mmHg
- Increase in lung infiltrates >50% during the previous 24–48 hours
- Alteration I n consciousness
- Multiple organ dysfunction
- Age >65 years
- Any of the above mentioned comorbidities

All units of transfused convalescent plasma had an Ig-G antibody titer ≥1:400. Levels of IgG anti-SARS-Cov2 were tested in all units by means of the test ELISA COVIDAR IgG, (Instituto Leloir, Argentina). This test utilizes the trimer of native protein S and a receptor binding domain as antigens, obtained by recombinant DNA techniques produced in human cells. The infused volume per unit was 200–250 ml.

Initial severity of illness was assessed according to the site of admission: general ward, Intensive Care Unit (ICU), and ICU admission with requirement of mechanical ventilation (ICU-VM). The main outcome variable was 28-day mortality. Deaths due to COVID-19 were confirmed on patient death certificates.

Statistical analysis: Continuous variables were expressed as mean ± standard deviation (SD) or median, [0.25–0.75] percentiles. Categorical variables were expressed as percentages. Differences between survivors and nonsurvivors, and between patients who received plasma or not, were analyzed with chi-square, t, or Mann-Whitney U-tests, as appropriate.

To identify independent predictors of 28-day mortality, variables differing between survivors and nonsurvivors with a *p* value <0.10 were entered into a multivariable regression model, using a forward stepwise analysis. The model was constructed in one block with all variables at the same level. Age was introduced as a continuous variable; site of admission as an ordinal variable using admission at general ward as reference group. Gender, risk factors and plasma administration were introduced as categorical variables. Adjusted risks were expressed as odd ratios (OR) and confidence intervals of 95% [CI95%]. A two-tailed p value <0.05 was considered significant.

Data were analyzed with SSPS-21 (Amonk, NY, US).

This study was approved by the Central Ethics Committee of the Ministry of Health of Buenos Aires Province (Expedient 2020–14965594). The resolution 103/2017 of the Ministry of Health of the Province of Buenos Aires establishes the obligation of registration and accreditation of all the Institutional Ethics Committees at the Central Ethics Committee of the Ministry of Health the Province of Buenos Aires; which is not associated with any institution or organization except the same Ministry, as it is the Ethics Committee of the said body, and evaluates all projects developed by institutions of the Ministry.

In the protocol of the present study, the Central Ethics Committee acts as an Institutional Evaluation Committee in use of the powers provided for by Decree 3385/08 as a research project, in which the Ministry of Health of the Province of Buenos Aires acts both as sponsor and center.

The Central Committee established that this observational study had an adequate risk-benefit ratio and requested the anonymization of data.

The administration of convalescent plasma required signed consent from each patient or legal representative, according to CROCPD-BA regulations (Expedient 2919/2123/2020).

## Results

During the study period, 3,529 patients with COVID-19 pneumonia were admitted to 215 hospitals. Epidemiological data of the entire group and comparisons between survivors and nonsurvivors are shown in Table 1. Briefly, this was a predominantly male population, aged 62±17 years, with arterial hypertension and diabetes as main comorbidities. With respect to disease severity, 51.4% were admitted to the ward, 27.1% to the ICU without mechanical ventilation need, and 21.7% to the ICU, with mechanical ventilation requirement (ICU-MV).

**Table 1:** Characteristics of the entire group, and comparison between survivors and nonsurvivors.

	All patients	Survivors	Nonsurvivors	P value
	n 3529	n 2298 (65.1%)	n 1231 (34.9%)	
Age (years)	62 ± 17	58 ± 17	69 ± 15	< 0.001
Gender (male)	2147 (60.8)	1419 (61.7)	718 (59.1)	0.130
Number of comorbidities				P < 0.001
0	1409 (39.9)	1028 (44.7)	381 (31.0)	
1	939 (26.6)	585 (25.5)	354 (28.8)	
2	683 (19.4)	425 (18.5)	258 (21.0)	
≥ 3	498 (14.1)	260 (11.3)	238 (19.3)	
Mean of comorbidities	1.20 ± 1.21	1.09 ± 1.18	1.39 ± 1.23	< 0.001
Arterial hypertension	1256 (35.6)	720 (31.3)	536 (43.5)	< 0.001
Diabetes	756 (21.4)	454 (19.8)	302 (24.5)	0.001
Preexistent cardiovascular disease	366 (10.4)	180 (7.8)	186 (15.1)	<0.001
Chronic obstructive pulmonary disease	268 (7.6)	161(7.0)	107(8.7)	0.072
Immunodeficiency	79 (2.2)	46 (2.0)	33 (2.7)	0.194
Site of admission				
General ward	1815 (57.4)	1337 (58.2)	478 (38.8)	<0.001
Intensive Care Unit	957 (27.1)	669 (29.1)	288 (23.4)	<0.001
Intensive care unit requiring mechanical ventilation	757 (21.7)	292 (12.7)	465 (37.8)	<0.001
Administration of convalescent plasma	868 (24.6)	647 (28.2)	221 (18.0)	<0.001
Length of ICU stay (days)	10 [5–19]	13 [6–24]	8 [4–14]	<0.001

Twenty-eight-day mortality was 34.9% for the entire group; and respectively, for ward, ICU and ICU-MV patients was 26.3%, 30.1% and 61.4%. Survivors were significantly younger, had less comorbidities, lower admission to the ICU, and had received plasma more frequently.

Convalescent plasma was administered to 868 patients (24.6%) (Table 2). Compared to the remaining 2,661, this group was composed of younger and predominantly male patients, with higher prevalence of arterial hypertension, diabetes, and higher ICU admission. The rate of mechanical ventilation use was similar in both groups.

**Table 2:** Characteristics of patients receiving and non-receiving convalescent plasma.

	No Plasma	Plasma	P value
	n 2661 (75.4%)	n 868 (24.6%)	
Age (years)	64 ± 17	56 ± 13	< 0.001
Gender (male)	1547 (58.1)	600 (69.1)	< 0.001
Number of comorbidities			P < 0.001
0	1128 (42.4)	281 (32.3)	
1	660 (24.2)	279 (32.1)	
2	495 (18.6)	188 (21.7)	
≥ 3	378 (14.2)	120 (13.8)	
Mean of comorbidities	1.11 ± 1.23	1.55 ± 1.06	< 0.001
Arterial hypertension	914 (34.3)	342 (39.4)	0.007
Diabetes	532 (20.0)	224 (25.8)	< 0.001
Preexistent cardiovascular disease	282 (10.6)	84 (9.7)	0.440
Chronic obstructive pulmonary disease	201 (8.7)	67 (7.7)	0.873
Immunodeficiency	59 (2.2)	20 (2.3)	0.880
Site of admission			
General ward	1409 (53)	406 (46.8)	0.002
Intensive Care Unit	677 (25.4)	280 (32.3)	< 0.001
Intensive care unit requiring mechanical ventilation	575 (21.6)	182 (21.0)	0.690
28-day mortality	1010 (38.0)	221 (25.5)	< 0.001
Length of ICU stay (days)	10 [4–17]	12 [7–18]	<0.001

Twenty-eight-day unadjusted mortality was lower in the entire group of patients receiving convalescent plasma, compared to those who had not (25.5% vs. 38.0%; OR 0.59 [0.47–0.66], p<0.001). The effect of plasma was more evident in patients in the ward (28-day mortality of 14.0% vs. 29.9% in those not receiving plasma; OR 0.38 [0.28–0.52], p<0.001), and in the ICU-MV patients (50.0% vs. 65.0%; OR 0.54 [0.38–0.75], p<0.001). In patients in the ICU who did not require MV the administration of plasma had no effect on mortality (26.1% vs. 31.8%; OR 0.76 [0.56–1.03], p = 0.081) (S1 Table in S1 File).

Logistic regression analysis identified age, ICU admission with and without MV, diabetes and preexistent cardiovascular disease as independent predictors of 28-day mortality, while hypertension and COPD were not independent predictors. Convalescent plasma administration was associated with decreased mortality (Table 3).

**Table 3:** Independent predictors of 28-day mortality, as identifies with logistic regression.

*Variables in the Equation*	B	SE	OR	95% C.I.	P value
Admission to the ICU (vs. admission to the ward)	0.26	0.09	1.30	1.08–1.56	0.006
Admission to the ICU with MV requirement (vs. admission to the ward)	1.75	0.10	5.73	4.71–7.00	< 0.001
Preexistent cardiovascular disease (yes/no)	0.38	0.12	1.46	1.14–1.85	0.002
Diabetes (yes/no)	0.29	0.09	1.33	1.11–1.60	0.002
Age (per year)	0.045	0.003	1.05	1.04–1.05	< 0.001
Administration of convalescent plasma (yes/no)	-0.31	0.10	0.73	0.60–0.89	0.002
*Variables not in the Equation*					
Hypertension					0.179
COPD					0.449

Abbreviations. ICU (Intensive Care Unit); MV (mechanical ventilation).

## Discussion

The main finding of our study was that older age, diabetes and antecedents of cardiovascular disease were independent risk factors for 28-day mortality for COVID-19 pneumonia, while the administration of convalescent plasma acted as a protective factor. This effect was more evident in less sick patients—those admitted to the general ward.

In this study, the global mortality of 34.6% was higher than the 21–28% shown in observational studies [20–23] which can be ascribed to a different patient case-mix. The proportion of patients admitted to the ICU was 42.6%, of which 21.6% required mechanical ventilation on admission. These figures are notably higher than those reported by two studies from Spain (respectively for each: n = 15,111 and 4,035, with ICU admission of 8.3% and 18%; and mortality of 21% and 28%); United States (n = 11,721, ICU admission of 19.9%, and mortality of 21.4%), and United Kingdom (n = 20,133, ICU admission 16.8%, and mortality of 26%) [20–23].

The efficacy of convalescent plasma in COVID-19 has been subject to much debate, due to the lack of a clinical trial with sufficient power to confirm it. For example, a study carried out in Wuhan was prematurely terminated due to the end of the pandemic, although significant clinical improvement was observed in patients with severe disease [10]. Likewise, a study from The Netherlands was stopped because 79% of patients already had high titers of neutralizing antibodies before receiving convalescent plasma [24]. A recent clinical trial from India which excluded critically ill patients did not find any clinical benefit. However, these results might be ascribed to the absence of neutralizing antibodies or to titers lower than 1:80 in 27% and 45% of convalescent plasma units, respectively [25]. Moreover, 86% of patients in the plasma subgroup had detectable neutralizing antibodies on enrollment; so it is uncertain if the intervention would have been efficacious.

Conversely, two small clinical trials demonstrated a significant decrease in mortality: in a study from Spain (n = 81) including severely ill patients, mortality in the convalescent plasma subgroup was 0% vs. 9.3% in the control, and in an Iraqi study (n = 49), it was 4.8% vs. 28.5%, respectively [26, 27].

Many observational studies support a probable efficacy of convalescent plasma. For example, a case-control study from China (including 138 cases and 1,568 controls) reported 2.2% mortality for the convalescent plasma subgroup, versus 4.1% for the control [28]. Furthermore, in a case-control study from the US including non-ventilated patients, 14-day mortality was 12.8% in the subgroup that had received convalescent plasma, vs. 24.4% in the control [29]. Similar results were reported in a matched case-control study, also from the US (136 cases, 251 controls), which showed lower mortality in patients receiving early administration of convalescent plasma with high titers of antibodies: 1.2% vs 8.9% [12]. Finally, the large case-series from the Mayo Clinic (n = 35,322) showed a relative risk of 30-day mortality of 0.77 [0.63–0.94] among patients transfused with plasma units of high antibody titers, compared to those transfused with low titers [14].

Our study develops a different approach to this very relevant issue. We analyzed a cohort of 3,529 unselected, consecutive patients with COVID-19 pneumonia, of whom 868 received convalescent plasma; its administration was evaluated as any other prognostic variable for mortality. We observed an independent, favorable effect on survival. Although the nature of our study was observational, it was carried out using a robust database composed of observations prospectively collected, within the framework of a pre-established government program. Other independent predictors of mortality were age, diabetes and cardiovascular disease, similar to current literature on the topic [22, 30–33].

This effect of convalescent plasma was more pronounced in less severe patients—those admitted to the ward, suggesting the importance of timely administration. Even though age >65 was one inclusion criterion for receiving convalescent plasma, surprisingly, those who received it were, in fact, younger. We cannot discard selection bias of physicians prescribing a seemingly promising therapy to patients with greater chances of responding to it. Nevertheless, older age was an independent predictor of mortality, as expected [22, 33].

The main limitation of this study is the lack of randomized assignment of convalescent plasma administration. A matching of cases receiving convalescent plasma with similar controls could not be done because of the type of data recorded in the register. It is thus possible that unmeasured confounders might have influenced the results, such as other risk factors or treatments. Notwithstanding this, the retrospective cohort design has already been applied for the analysis of the effect of convalescent plasma on COVID-19 [14]. Regarding possible differences in outcomes related to the adoption of specific therapies for COVID-19, the Ministry of Health of the Province of Buenos Aires has issued recommendations for the treatment of COVID-19, and encourages compliance with them. Since severity of illness on admission could not be evaluated with an established score, hence misclassification of patients might have occurred. The use of severity of illness on admission as a surrogate of acuity. However, our approach has already been utilized [3]. It is possible that convalescent plasma might be efficacious in particular patient subgroups. In this way, observational studies might help to identify populations in which this therapy might be beneficial, such as in patients with less severe disease but with high potential of clinical deterioration, or in those with short duration of symptoms. The duration of symptoms before convalescent plasma administration was not available in our cohort. Although a possible key determinant of convalescent plasma effect, previous symptom duration is difficult to ascertain and might be subjected to recall bias. A more detailed analysis of the clinical variables collected could not be done, because of the type of data recorded in the register. Finally, the reason why assistant physicians chose not to administer convalescent plasma to patients with COVID-19 pneumonia fulfilling the inclusion criteria are unknown, but we speculate that some physicians might have felt uncomfortable with prescribing an experimental treatment to their patients.

## Conclusions

Our study suggests that the administration of convalescent plasma in COVID-19 pneumonia might be associated with better outcomes. Large, well-designed clinical trials are required to confirm these findings.

## Supporting information

S1 File (DOCX)
