# Supplementary material for: Risk factors for COVID-19 mortality: The effect of convalescent plasma administration
Source: PLoS One. 2021 Apr 29;16(4):e0250386. doi: 10.1371/journal.pone.0250386 (PMC8084206; doi:10.1371/journal.pone.0250386)
Supplement: S1 File — (DOCX) [file pone.0250386.s001.docx]

Early in the COVID-19 pandemic, the Ministry of Health of the Province of Buenos Aires, Argentina created the Centralized Registry of Convalescent Plasma Donors (*CROCPD-BA*).

Possible donors were contacted from patients recovered from COVID-19, on the basis of solidarity and social responsibility principles. After they expressed the will to donate convalescent plasma, donors were scheduled to the centers authorized by the CROCPD-BA, where they signed a written informed consent.

A negative RT-PCR test for SARS-CoV-2 was a prerequisite for donating plasma.

Plasma was obtained by the apheresis method.

Antibody titrating was performed in the Immunoserology Section of Central Laboratory of the Children’s Hospital in La Plata, Buenos Aires, Argentina. All units of transfused convalescent plasma had an Ig-G antibody titer ≥1:400.

During the study period (6/1/20 to 7/31/20) 974 units were transfused (1,12 units per patient on average). The infused volume per unit was 200-250 ml. Dosing was estimated according to weight; patients with <70kg received 1 unit, and those >70 kg received 2 units.

**S1 Table.** Effect of convalescent plasma on 28-day mortality on the entire population, and on the different subgroups according to patient site of admission.

| Site of admission | Total  Number of patients | Not receiving plasma | Receiving plasma | Nonsurvivors  Not receiving Plasma | Nonsurvivors  Receiving  Plasma | p | Unadjusted  OR (95%CI)  for mortality |
| --- | --- | --- | --- | --- | --- | --- | --- |
| General Ward | 1815 | 1409 (77.6) | 406  (22.4) | 421  (29.9) | 57  (14.0) | < 0.001 | 0.38 [0.28-0.62] |
| ICU | 957 | 677  (70.7) | 280  (29.3) | 125  (31.8) | 73  (26.1) | 0.081 | 0.76 [0.56-1.04] |
| ICU-MV | 757 | 575  (75.9) | 182  (24.1) | 374  (65.0) | 91  (50.0) | < 0.001 | 0.54 [0.38-0.75] |
| All patients | 3529 | 2661  (75.4) | 868  (25.4) | 1010  (38.0) | 221  (25.5) | < 0.001 | 0.56 [0.47-0.66] |

All results are expressed as n, (%), unless specified

ICU: Intensive Care Unit

ICU MV: admitted to the ICU and requiring Mechanical ventilation
